# Relevance of Membrane Contact Sites in Cancer Progression

**DOI:** 10.3389/fcell.2020.622215

**Published:** 2021-01-12

**Authors:** Aurora Gil-Hernández, Miguel Arroyo-Campuzano, Arturo Simoni-Nieves, Cecilia Zazueta, Luis Enrique Gomez-Quiroz, Alejandro Silva-Palacios

**Affiliations:** ^1^Departamento de Biomedicina Cardiovascular, Instituto Nacional de Cardiología Ignacio Chávez, Mexico City, Mexico; ^2^Departamento de Ciencias de la Salud, Universidad Autónoma Metropolitana-Iztapalapa, Mexico City, Mexico

**Keywords:** membrane contact sites, metastasis, calcium signaling, lipid signaling, cancer progression

## Abstract

Membrane contact sites (MCS) are typically defined as areas of proximity between heterologous or homologous membranes characterized by specific proteins. The study of MCS is considered as an emergent field that shows how crucial organelle interactions are in cell physiology. MCS regulate a myriad of physiological processes such as apoptosis, calcium, and lipid signaling, just to name a few. The membranal interactions between the endoplasmic reticulum (ER)–mitochondria, the ER–plasma membrane, and the vesicular traffic have received special attention in recent years, particularly in cancer research, in which it has been proposed that MCS regulate tumor metabolism and fate, contributing to their progression. However, as the therapeutic or diagnostic potential of MCS has not been fully revisited, in this review, we provide recent information on MCS relevance on calcium and lipid signaling in cancer cells and on its role in tumor progression. We also describe some proteins associated with MCS, like CERT, STIM1, VDAC, and Orai, that impact on cancer progression and that could be a possible diagnostic marker. Overall, these information might contribute to the understanding of the complex biology of cancer cells.

## Introduction

Cancer is a serious public health problem worldwide (Henley et al., 2020a); the most common types among women are lung, breast, and colorectal tumor, whereas lung, prostate, and colorectal cancer prevails in men. Globally, 25% of such deaths were from the lung, 9% colorectal, 7% from breast, and 5% from prostate cancer (Henley et al., 2020b).

Breast cancer is the main diagnosis among young women (Rugo, 2019). The risk of recurrence remains latent up to 15 years after adjuvant therapy, as there is an association between progression and risk of metastasis. Breast stages I–III tumors and regional lymph nodes are characterized by 5–10 years survival rates (Liu X. et al., 2019), whereas patients with stage IV metastatic breast cancer have 5 years survival rates below 25%. On average, 5–10% of patients are classified as with stage IV disease at initial diagnosis, but 20–30% of stage I–III patients eventually progress to metastatic disease (Liu X. et al., 2019). Even if the cancer mortality rates decreased by 15% from 2007 to 2017, breast cancer caused 20.7 deaths per 100,000 in women (Henley et al., 2020b), and 1,735,350 new cases were reported in 2018. According to the National Institutes of Health, up to 23.6 million total cases are expected in 2030; therefore, breast cancer will remain one of the deadliest diseases for individuals^1^.

Cancer groups have around 100 highly heterogeneous kinds of diseases, with variable morphological and biological characteristics; hence, the different clinical behaviors and responses to treatment (So et al., 2019) complicate chemoresistance. The classification of cancer is based on its morphology, histological grade (level of differentiation/growth pattern), immunohistochemical subtype, as well as gene expression profiling (Golub et al., 1999; So et al., 2019; Tsang and Tse, 2020). So, to establish the clinical management of cancer in patients, it is essential to understand the underlying biological process that sustains the metabolic and signaling requirements for adequate tumor dynamism, including diffusion or active transport through the cytoplasm, vesicular traffic, and contact site biology (Prinz et al., 2020).

**FIGURE 1:**
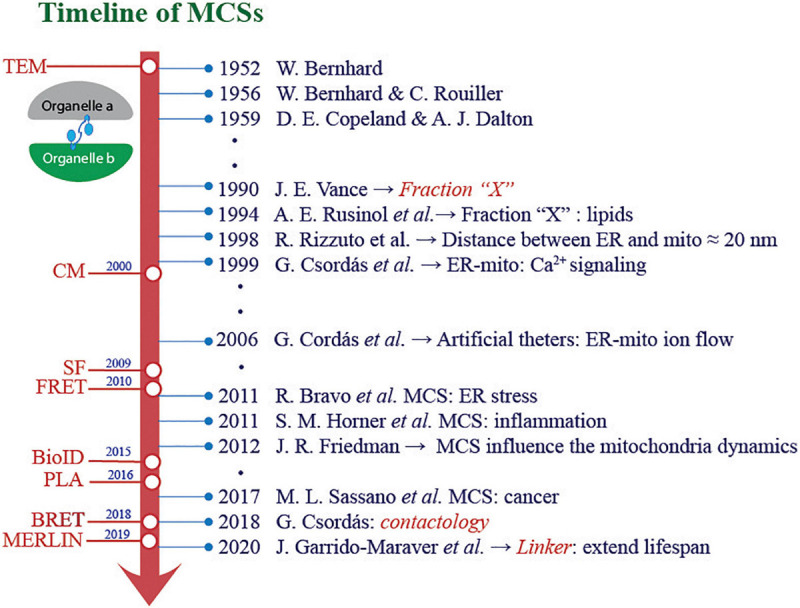
Scientific and technological advances in the study of membrane contact sites. The knowledge about organelle contacts and their role in different pathophysiological conditions has experienced a great “boom.” In harmony with these advances, significant progress has been made in optimizing methodology strategies to study and comprehend the interaction between organelles. TEM, transmission electronic microscopy; CM, confocal microscopy; SF, subcellular fractionation; FRET, fluorescence resonance assembly transfer; BioID, proximity-dependent biotin identification; PLA, proximity ligation assay; BRET, bioluminescence resonance energy transfer; MERLIN, mitochondria–endoplasmic reticulum length indicator nanosensor.

Membrane contact sites (MCS) contribute to maintain the function of the interacting organelles and to preserve cell homeostasis (Prinz et al., 2020). Electron microscopy made these connections evident decades ago (Bernhard and Rouiller, 1956; Copeland and Dalton, 1959), and lately, improved spatial and temporal resolution imaging has led to track organelle dynamics over time, revealing the extent to which many of them are closely related (Wu et al., 2018; Figure 1). The best-described membrane contacts involve endoplasmic reticulum (ER)–mitochondria, ER–plasma membrane (PM), ER–Golgi, ER–peroxisome, and ER–lipid droplets (LD) interactions; however, MCS also include LD–peroxisome, mitochondria–vacuole, mitochondria–PM, mitochondria–LD, and mitochondria–peroxisome (Scorrano et al., 2019).

MCS are not only considered as regulatory sites for lipid metabolism and intracellular calcium [(Ca^2+^)_i_] homeostasis but they also modulate the organelle distribution and structure (Prinz, 2014). Some functions and interactions of MCS have been identified and described to regulate cancer cell metabolism during disease progression. For example, hexokinase 2 overexpression, which is related to the stages of cancer progression, acquisition of invasive and metastatic capabilities, and poor prognosis (Patra et al., 2013), has been located in the mitochondria-associated membranes (MAMs), and its displacement induces mitochondrial Ca^2+^ [(Ca^2+^)_mt_] overload (Ciscato et al., 2020).

In the present work, we reviewed in detail the state-of-the-art of MCS-mediated Ca^2+^ and lipid signaling in cancer, specifically the interactions between ER–PM, ER–mitochondria, and ER–Golgi. Besides this, we describe the involvement of these contact sites in cancer progression, and finally, we address MCS as possible candidates for therapy.

## Membrane Contact Sites

MCS are defined as areas where two different organelles physically interact without merging, and therefore the identity of each of them is preserved (Jing et al., 2020; Venditti et al., 2020). MCS are dynamic and heterogeneous structures composed of different proteins that act as a bridge between the two membranes, exerting a binding force, that might have additional functions in the cell (Eisenberg-Bord et al., 2016; Peretti et al., 2020). ER, which occupies the largest surface area in the cell, is involved in the formation of many MCS with other organelles (Venditti et al., 2019, 2020). ER contact sites allow the direct exchange of macromolecules and serve as a platform for the recruitment of machinery that regulates biogenesis, division, and trafficking of organelles (Lee et al., 2020). Among the broad processes taking place at such interface are Ca^2+^ homeostasis, lipid signaling, and organelle remodeling (Jing et al., 2020) as well as mitochondrial fusion/fission, autophagy, apoptosis, reactive oxygen species (ROS) signaling, and unfolded protein response (van Vliet et al., 2014; Silva-Palacios et al., 2020).

### Endoplasmic Reticulum–Plasma Membrane

ER–PM contacts were first described by electron microscopy in muscle cells (Porter and Palade, 1957) and eventually in many other cell types (Okeke et al., 2016). Their abundance and morphology vary from one cell to another and can be modulated by its functional status. The ER–PM contact sites are represented by both small focal contacts and large cisterns with a gap between ER and PM in the range of 10–30 nm (Orci et al., 2009; Fernández-Busnadiego et al., 2015) that allows the direct interaction of protein and lipid components in both membranes.

Currently, it is well known that the oxysterol-binding protein (OSBP)-related protein (ORP) is the major lipid transporter between ER and PM contacts, specifically of phosphatidylserine (PS) and phosphatidylinositol 4-phosphate (PI4P). Similarly, OSBP counteracts the transport of sterols and PI4P in the ER–Golgi contact (see below), while Osh3 (OSBP homolog in yeast) regulates the metabolism of phosphoinositol (PI) (Fernández-Busnadiego, 2016). Recently, it was reported that a robust exchange of sterols at the ER–PM contact site in yeasts lacking tether proteins (including E-synaptotagmin and the vesicle-associated membrane protein, VAMP). These sites act as an interface for the regulation and integration of lipid synthesis pathways to maintain plasma membrane composition and integrity. Loss of ER–PM contacts in yeast has been associated with low levels of PS, phosphatidylcholine, and phosphatidic acid and with the disruption of membrane dynamics (Quon and Beh, 2016; Quon et al., 2018). The vesicle-associated protein (VAP) in mammals neither appears to be strictly necessary for the maintenance of ER–PM contact, probably because other transmembrane proteins of the endoplasmic reticulum are involved in the tethering complex (Chung et al., 2015; Lees et al., 2017). The ER–PM contact sites in non-muscular cells remained poorly described until recently, when the discovery of the stromal interaction molecule 1 system (STIM/Orai1) revealed that these contacts mediate calcium input through the store-operated Ca^2+^ entry system (SOCE) (see below) into all metazoan cells (Collado and Fernández-Busnadiego, 2017). In addition, inositol 1,4,5-trisphosphate receptor (IP_3_R) might form a complex with STIM1 and improve the SOCE pathway cation input (Paknejad and Hite, 2018; Sampieri et al., 2018).

### Endoplasmic Reticulum–Mitochondria

MAMs are specific MCS between the ER and the mitochondria, which participate in different cellular functions (Peretti et al., 2020). The average distance between both organelles varies from 10 to 60 nm. In this sense, several studies indicate that 9–16 nm is enough distance to tether the outer mitochondrial membrane (OMM) to the smooth ER, while a space of 20 nm has been observed between the OMM and the rough ER (Achleitner et al., 1999; Csordás et al., 2006; Giacomello and Pellegrini, 2016; Simmen and Herrera-Cruz, 2018; Wu et al., 2018).

It has been reported that distances between MAMs greater than 30 nm are required for Ca^2+^ transport in cardiomyocytes (Sharma et al., 2000). The MAM tethering axis includes the IP_3_R in the ER which is pivotal for communication and interaction with the voltage-dependent anion channel (VDAC), which is located at the OMM and is responsible for the release of adenosine triphosphate (ATP) from the mitochondria to the cytosol (Fang and Maldonado, 2018). It is relevant to note that isoform 1 of IP_3_R is crucial for calcium exchange with the mitochondria (Bartok et al., 2019).

The interaction between IP_3_R and VDAC is mediated by glucose-regulated protein 75 (Grp75), which participates in calcium exchange and stabilizes the membrane contact site (Szabadkai et al., 2006; Patergnani et al., 2011). Mitofusin 2 (Mfn2), mostly located in MAM (de Brito and Scorrano, 2008), also regulates calcium transport in ER–mitochondria contacts. MAMs convey calcium signaling between the IP_3_R and the mitochondrial calcium uniporter (MCU), the channel responsible for Ca^2+^ uptake into the mitochondrial matrix. Other recently described proteins that maintain MAM integrity are DJ-1 (oncoprotein encoded in PARK7 gene) (Xu et al., 2018; Liu Y. et al., 2019; Basso et al., 2020) and PDZ domain-containing protein 8 (a synaptotagmin-like mitochondrial lipid-binding protein domain-containing ER transmembrane protein) that has been implicated in ER-dependent mitochondrial calcium homeostasis (Hirabayashi et al., 2017; Elbaz-Alon et al., 2020), while pannexin 2 has been reported to sensitize cancer cells to apoptotic stimuli (Le Vasseur et al., 2019). In triple-negative breast cancer (TNBC), MCU silencing disturbs calcium uptake, enhancing alternative pathways such as SOCE, whereas the reduction of mitochondrial calcium levels inhibits cell migration in cancer cell lines. Depletion of MCU expression also reduces ROS production and deregulates hypoxia-inducible factor 1 alpha, diminishing cancer progression (Tosatto et al., 2016).

### Endoplasmic Reticulum–Trans Golgi Network

The Golgi complex is part of the cytoplasmic endomembrane system, which is normally found adjacent to the nucleus (Li et al., 2019). Transmission electron microscopy analysis revealed that the Golgi forms stacks consisting of eight-storey cisterns placed parallel to each other (Donizy and Marczuk, 2019). The Golgi complex is divided into three compartments: (1) the *cis*-Golgi network (near the ER and receiving its output), (2) the middle part, and (3) the *trans*-Golgi network (TGN, near the PM that sends vesicle-dependent and vesicle-independent molecules to different destinations) (Witkos and Lowe, 2016). This peculiar structure actively participates in the traffic of proteins and lipids into the cell and regulates post-translational modification (De Matteis and Rega, 2015; Howley and Howe, 2018). The phospholipid composition of the ER and the *trans*-Golgi membrane differ significantly, the cytosolic side of the ER membrane is slightly charged, and phospholipids are enriched in monounsaturated chains, while the TGN membrane is enriched with sphingolipids and with negatively charged lipids such as PS, PI, and PI4P on its cytosolic side (Mesmin et al., 2019).

The ER and Golgi network is related to the molecular traffic of vesicular and non-vesicular routes (Lev, 2010, 2012; Wu et al., 2018). Specifically, the VAMP-associated protein is involved in the formation of MCS between ER and almost all organelles (Murphy and Levine, 2016). At ER–Golgi sites, VAPs provide contacts with FFAT motifs [two phenylalanine (FF) in an acid tract] of three different proteins involved in lipid transfer such as Nir2 (important for PI transfer from the ER to the PM), ceramide transferase (CERT, responsible for ceramide transfer between ER and TGN) (Hanada et al., 2003), and ORP protein, which exclusively transfers cholesterol and PI4P but also acts as tethers agent (De Matteis and Rega, 2015).

## Regulated Cellular Processes at Contact Sites and Their Relation to Cancer Progression

### Calcium Signaling

MCSs allows the efficient transfer of metabolites between compartments, particularly of calcium. The cytosol calcium concentrations are ∼100 nM (Marchi et al., 2020), whereas in the ER lumen [(Ca^2+^)_ER_], it reaches 100–800 μM (Burdakov et al., 2005; Zhai et al., 2020).

The role of calcium signaling in cancer progression has been discussed in detail recently (Marchi and Pinton, 2016; Bong and Monteith, 2018). Calcium regulates cellular processes such as proliferation, migration, and resistance (among others), contributing to the development of a malignant phenotype that is essential and continuously rewired at all stages of carcinogenesis (Morciano et al., 2018). This scenario seems to be caused by the uneven regulation of pumps and channels and to the consequent alteration in calcium concentration (Dang et al., 2017; So et al., 2019; Makena and Rao, 2020; Yang et al., 2020). It has been proposed that changes in the distance and morphology of the mitochondrial tether can dramatically alter calcium homeostasis and cell function (Simoes et al., 2020); therefore, it is imperative to understand the regulation of this cation by MCS in cancer.

#### Store-Operated Ca^2+^ Entry

Store-operated Ca^2+^ entry (SOCE) is an ancient and ubiquitous Ca^2+^ signaling pathway discovered decades ago (PutneyJr., 1986), the main components of which are the stromal interaction molecule 1 (STIM1) and Orai1 (Roos et al., 2005; Zhang et al., 2005; Feske et al., 2006). The depletion of the Ca^2+^ store directly results in the activation of the Ca^2+^ channel in the plasma membrane (PutneyJr., 1986), whereas SOCE is activated by membrane receptors or by pharmacological manipulations that empty the ER intracellular Ca^2+^ stores (Xie J. et al., 2016).

**FIGURE 2:**
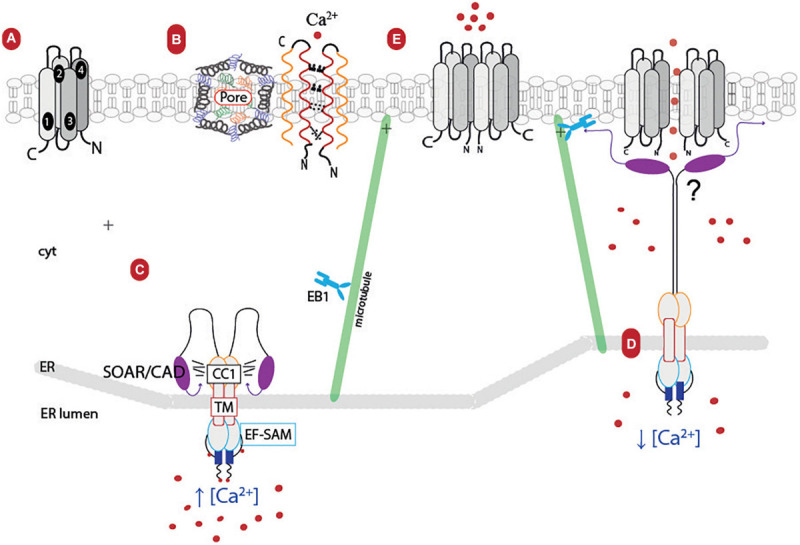
Cartoon model of ER–PM junction (store-operated Ca^2+^ entry system pathway). **(A)** Orai’s monomeric structure: TM1-4 and its C- and N-terminal endings facing the cytosol. **(B)** Orai channel structure: The channel is conformed by six subunits (monomers) which interact with each other due to the hydrophobic cluster (E106, V102, F99, R91) in their respective TM1. These interactions leave the ion pore at the center of the channel, and it is also stabilized by the TM1–TM2 turret, causing the channel’s selectivity. **(C)** STIM1 resting state: This protein seems to dimerize in basal conditions. STIM1 monomer’s structure consists of the EF-SAM calcium sensor, facing the ER-lumen, the TM domain, and the CC1, SOAR/CAD segments oriented to the cytosol. The STIM1 monomer in the presence of calcium is folded into the cytosol domain due to hydrophobic interactions between the coiled coils. **(D)** STIM1 extended state: STIM1 interacts with EB1 protein, helping to remodel the ER and forming STIM1 clusters near the PM; its polybasic tail also seems to interact with the PM. When there is a Ca^2+^ depletion in the ER lumen, STIM1 suffers a conformational change, and both monomers release its SOAR/CAD segments, which, in turn, interact with Orai. **(E)** Possible activation of Orai: STIM1 could contribute to either rotate Orai’s helices or help the subunits’ outward movement. Orai loses its high permeability and allows the entry of extracellular calcium. TM, transmembrane helices; EF-SAM, EF hand 1, non-canonical ER hand 2, and sterile alfa motile; CC1, coiled coil 1; SOAR/CAD, Stim1-Orai1 activating region/CRAC activation domain; EB1, microtubule plus-end tracking of end binding protein 1; cyt, cytosol; ER, endoplasmic reticulum; PM, plasma membrane.

STIM proteins (in humans 1 and 2) are single-pass membrane proteins located in the ER membrane that, after ER Ca^2+^ depletion, regulate the Orai channel (Hou et al., 2020). Structurally, all STIM1 monomers contain an N-terminal signal peptide, a canonical Ca^2+^-binding EF 1 hand, a non-Ca^2+^-binding EF 2 hand, and a sterile α-motif (SAM) domain in the ER luminal region (known as ER-SAM) (Dziadek and Johnstone, 2007; Schober et al., 2019). The C-terminus domain located in the cytosolic side of the ER membrane is characterized by the coiled-coil 1 (CC1) segments, a STIM-Orai-activating region (SOAR) or calcium release-activated calcium (CRAC) activation domain (CAD), and the motif S/TxIP which are the components of the Orai1 activation small fragment (Chen Y. T. et al., 2013; Stathopulos et al., 2013; Rathner et al., 2018; Figure 2).

When the [Ca^2+^]_ER_ decreases, the cation dissociates from the different binding sites in the EF-SAM, producing a conformational change in this region (Zheng et al., 2011). That this, at rest, the EF-SAM region of STIM1 is monomers, while the cytosolic region of STIM1 are dimers. After activation, EF-SAM oligomerizes, which leads to conformational changes in its cytosolic region, elongating the SOAR/CAD segments (extended state) and the S/TxIP motif (Gudlur et al., 2018; Hirve et al., 2018). Thanks to this and its positively charged polybasic tail, STIM1 moves along the microtubules network interacting with the EB1 protein, generating clusters and elongation of the ER near the PM and favoring its union with Orai1 (Grigoriev et al., 2008) (reviewed in Ma et al., 2020).

#### Calcium Release-Activated Calcium Channel

Orai is the pore-forming protein of the calcium release-activated calcium (CRAC) channel; there are three human Orai (Orai1-3) proteins which consist of four transmembrane (TM) helices (TM1–4) with cytoplasmic N- and C-terminal domains (Hou et al., 2012; Stathopulos et al., 2013). The channel structure consists of six Orai subunits arranged as a hexamer with a central pore (Hou et al., 2012; Cai et al., 2016). TM2–4 forms concentric rings around the ion-conducting pore, surrounding the TM1 helices located at the boundary of the ion pore. A TM4 extension binds with a SOAR, promoting coupling between PM and ER and mediating intracellular Ca^2+^ mobilization (Park et al., 2009; Cai et al., 2018). Orai’s selectivity for calcium is determined by the mainly electronegative charged TM1–TM2 turret structure, which is stabilized by a VQLD motif and a lysine residue from the TM3 (K270 in *D. melanogaster*) and by the narrowing of the region near the Ca^2+^ binding site formed by the E106 residue (Yeung et al., 2018; Bulla et al., 2019; Hou et al., 2020).

It has been proposed that, after Ca^2+^ store depletion in ER, the CRAC channels interact both at their cytoplasmic N- and C-termini with the ER Ca^2+^ sensor protein STIM1, resulting in close coupling in the apposed sites at the ER–plasma membrane junctions (Figure 2). Specifically, the SOAR/CAD segment interacts with the Orai1 hexamer, producing the pore helix, which displaces six F99 residues and disrupts the V102-F99 hydrophobic segment that closes the channel, increasing port hydration and calcium conduction (Yamashita et al., 2017, 2020). Another hypothesis, derived from cryo-electron microscopy (3.3 Å resolution) studies in *D. melanogaster*’s mutant protein, suggests that Orai subunits are displaced, causing the repositioning of F171 (F99 in human Orai), widening the hydrophobic región, and promoting channel opening (Hou et al., 2020).

The SOCE pathway involvement in cancer progression is undeniable and might be a target for the study and treatment of this disease. Flourakis et al. (2010) demonstrated that SOCE is the main source of Ca^2+^ influx that triggers apoptotic cell death in human prostate cancer cells. Orai1 *knockdown* in cancer cells inhibits SOCE and protects these cells from apoptotic death (Flourakis et al., 2010). Also, the inhibition of STIM1 and Orai1 reduces cell migration and tumor metastasis in breast cancer cells by down-regulating the calcium-dependent focal adhesion pathway (Yang et al., 2009), an effect also seen in cervical cancer (Chen Y. F. et al., 2011, 2013) and hepatocarcinoma cells (Yang et al., 2013).

It has been shown that pharmacological upregulation of the SOCE pathway decreases glioblastoma tumor cell growth, activating Ca^2+^ entry and inhibiting the YAP/TAZ pathway (a key transcription factor that regulates tumor cell proliferation and aggressiveness) (Enzo et al., 2015). On the other hand, SOCE activates the extracellular signal-regulated kinase (ERK) signaling pathway, promoting cell proliferation and migration in melanoma cells (Umemura et al., 2014), while Orai1/STIM1 enhances Akt activity, contributing to *cis*-platin resistance in ovarian carcinoma cells (Schmidt et al., 2014). Recently, it was reported that the inhibition of STIM1/Orai1 interactions in TNBC cell lines with NO1, a fluorescent ligand for Sigma-2R (initially described as a cholesterol transport regulator), causes an alteration of the SOCE pathway, increasing apoptotic cell death and reducing the proliferation and migration rate of these cells (Cantonero et al., 2020).

Ineffective epithelial–mesenchymal transition (EMT) and metastasis suppression were observed in MDA-MB-231 and MCF-7 cell lines treated with transforming growth factor-β (TGF-β) in which STIM1 was *knocked-down*, proving a clue of the role of SOCE in TGF-β-induced cancer progression (Zhang et al., 2017). Furthermore, in very aggressive cancer cell lines (such as MDA-MB-231), STIM1 is overexpressed as compared to the MCF-7 cell line (Kulkarni et al., 2019).

On the other hand, deregulation of Orai by siRNA inhibition in cancer cell lines promotes deregulation of proteins involved in the cell cycle such as cyclin-D1 and cyclin-E, as well as overexpression of p53 and p21, together with an increase in [Ca^2+^]_*i*_, which collectively favor apoptosis (Faouzi et al., 2011). Besides these, Orai3 produces resistance to chemotherapeutic drugs through increased free calcium uptake, which leads to p53 inactivation (Hasna et al., 2018). In turn, silencing of Orai1 and 2 with SOCE chemical inhibitors and siRNAs inhibits calcium uptake and suppresses cancer cell proliferation, colony formation, and migration in association with inhibition of the Akt/mammalian target of rapamycin (mTOR)/nuclear factor κB (NF-κB) pathway (Singh et al., 2020). Orai can be inactivated through phosphorylation in its Ser-27, -30, or -34 residues. In MDA-MB-231 cell lines, it has been reported that overexpression of Orai1, as well as calcium–calmodulin-activated adenylyl cyclase type 8 (which interacts at phosphorylation sites), prevents the inactivation of Orai1, increasing calcium signaling and promoting cancer cell migration (Sanchez-Collado et al., 2019). In summary, these examples highlight the relevance of the ER–PM contact site both in calcium signaling and in other physiological pathways.

Clinically, STIM1 has been correlated with poor prognosis in breast (Yang et al., 2017), colorectal (Wang et al., 2015), and lung carcinoma (Zhan et al., 2015) because its overexpression in patients’ tissues is associated with increased tumor size, lymphatic metastases, and other factors. Orai3 is also overexpressed in several types of cancer such as esophageal and gastric carcinoma among others (Zhu et al., 2014; Xia et al., 2016; Wang L. et al., 2017).

In conclusion, [Ca^2+^]_i_ modulated by SOCE is a master physiological regulator that contributes to uncontrolled proliferation and malignant tumor progression (Sharma and Elble, 2020). We must recall that mitochondrial Ca^2+^ activates the Krebs cycle, apoptosis, and mitochondrial fission (Harper et al., 2020). In particular, the activity of α-ketoglutarate, isocitrate, and pyruvate dehydrogenases is controlled by the cation, increasing the ATP synthesis. However, cancer cells require a high glycolytic rate (Warburg effect) to maintain growth and tumor progression. Such metabolic switch has been associated with alterations in calcium signaling in MAMs (Bittremieux et al., 2016). Therefore, deregulation of Ca^2+^ import via MAMs could affect tumorigenesis through metabolism and cell death (Peretti et al., 2020).

**FIGURE 3:**
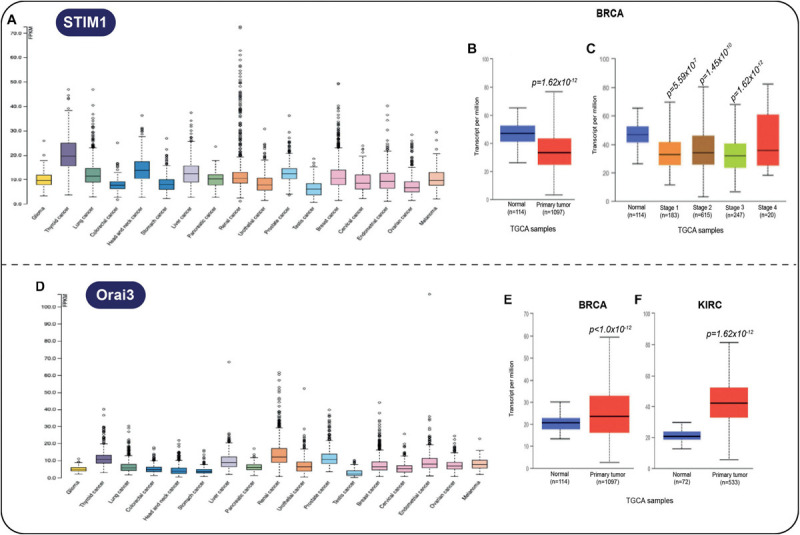
**(A)** *Stim1* gene product is elevated in several solid tumors. **(B)** *Stim1* gene expression in breast cancer (BRCA) vs. tissue samples (*p* = 1.62 × 10^− 12^) and **(C)** *Stim1* gene expression in all stages of BRCA vs. normal samples. **(D)** *Orai3* gene overexpression is associated with many cancers, in particular with **(E)** BRCA (*p* < 1.0 × 10^− 12^) and with **(F)** KIRC (*p* = 1.62 × 10^− 12^). All data were taken by UALCAN cancer database (Chandrashekar et al., 2017).

Further information of the expression profile of some MCS protein-related genes is included in the cancer genomic database The Cancer Genome Atlas (TCGA), which is integrated with clinical characteristics, including patient outcome in common cancer types. For example, the *Stim1* gene was shown to be elevated in many solid tumors (Figure 3A), but the gene product is not considered as prognostic. Interestingly, the expression of the *Stim1* gene is diminished in breast cancer (Figure 3B) and is practically comparable to normal tissue levels at all stages (Figure 3C). Regarding Orai, its expression increases (Figure 3D), particularly in breast (Figure 3E) and renal cancer (Figure 3F). These changes could be easily addressed by conventional methods such as qRT-PCR to confirm their prognostic relevance.

#### IP_3_R

Overexpression of IP_3_1 and 2 receptors has been related to apoptosis in different tissues and cancer cells (Assefa et al., 2004; Kopacek et al., 2009; Akl et al., 2013; Hudecova et al., 2016) and to compromised cell metabolism (particularly glycolytic and the mitochondrial bioenergetic) (Singh et al., 2017). Recently, it has been reported that down-regulation of IP_3_R3 correlates with a lower migration rate due to the modulation of Ca^2+^ signaling. Accordingly, overexpression of IP_3_R in cancer cells improves free calcium fluctuation and promotes efficient migration (Mound et al., 2017). Migration is also associated with cell morphology, i.e., when IP_3_R3 is silenced, a rounded shape is produced in highly invasive cancer cell lines, resulting in a low rate of adhesion and migration, as a consequence of Ca^2+^ oscillation and cytoskeletal changes driven through the ARHGAP18/RhoA/mDia1/FAK signaling pathway (Vautrin-Glabik et al., 2018). This silencing is also related to the induction of apoptosis in different cancer cell lines such as colorectal, ovarian, and clear cell renal carcinoma tumors (Rezuchova et al., 2019).

#### VDAC

On the other hand, the role of the mitochondrial protein VDAC in cancer is debated. For example, VDAC1 *knockdown* has been related to proliferation inhibition. Also, the VDAC inhibitor, JQ1, prevents the activity of bromodomain-containing proteins (BRD), including BRD4, that is enriched in patients with basal luminal breast cancer (Qi, 2014), suggesting that VDAC1 is relevant in cancer cell progression and is a hallmark of poor prognosis (Yang et al., 2019).

In this regard, Dr. Lev’s group recently identified that the combination of the synthetic drug JQ1 and the proteasome inhibitor bortezomib induces ferroptosis in multiple TNBC cells line *in vitro* and *in vivo*. The findings of this group indicate that combination therapies effectively decrease the size of the tumor and significantly prolong the survival of mice up to 80 days. Mechanistically, these therapies induce ferroptosis in correlation with the reduction of glutathione peroxidase 4, nuclear factor erythroid 2-related factor 2 (Nrf2), and glutathione metabolism. Therefore, drug combination might be a new therapeutic option for patients with triple-negative breast cancer, highlighting ferroptosis as a promising avenue for the treatment of TNBC (Verma et al., 2020). Relevant to this issue is the finding that progesterone induces VDAC and sarco(endo)plasmic reticulum Ca^2+^-ATPase (SERCA) expression, inhibiting the growth of MCF-7 cells (Azeez et al., 2018).

**FIGURE 4:**
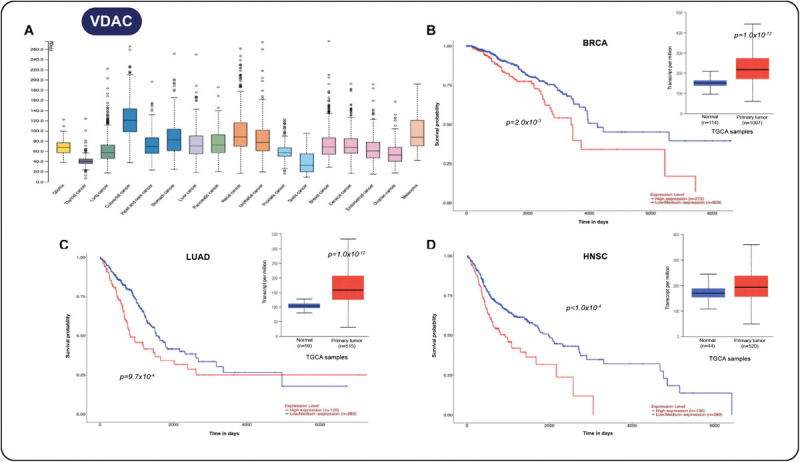
**(A)** Increment in voltage-dependent anion channel (VDAC) in several solid tumors. **(B)** Survival analysis for the *VDAC* gene and expression in breast cancer samples vs. normal tissue (*p* = 1.0 × 10^− 12^). **(C)** Survival analysis of VDAC gene and expression in lung adenocarcinoma vs. normal tissue samples (*p* = 1.0 × 10^− 12^). **(D)** Survival analysis and expression of VDAC in head and neck carcinoma samples vs. normal tissue samples (*p* = 1.0 × 10^− 12^). All data were taken from the UALCAN cancer database (Chandrashekar et al., 2017).

Using the TCGA database, we also detect VDAC overexpression in several solid tumors (Figure 4A). Notably, in breast cancer (Figure 4B), lung adenocarcinoma (Figure 4C), and head and neck carcinoma (Figure 4D), a close relationship exists between gene expression and poor survival outcome. By using the extraordinary resources based on “omics” data, more MCS-associated protein could be relevant in the clinical context; some of them might be useful for outcome prediction, particularly in the emerging “contactology science.”

#### Grp75

Grp75 (also known as mortalin or HSPA9) interacts with p53 (the master regulator of multiple cellular physiological processes such as apoptosis, senescence, cell cycle arrest, etc.), preventing its activation and therefore promoting cancer cell survival. In human breast cancer cells, treatment with the molecule embelin inhibits Grp75-p53 interaction, leading to the downregulation of Grp75 as long as metastatic signaling occurs, inhibiting mitochondrial fission and arresting growth (Nigam et al., 2015). Grp75 is also a metastatic hallmark in cancer. Interestingly, mortalin also increases in adjacent non-tumor cells, suggesting that such enrichment could be detected at the early stages of cancer (Jin et al., 2016).

#### PERK

The protein kinase RNA like ER kinase (PERK) is another tether in MAM extensions that could promote or suppress tumor progression in cancer cells. PERK triggers multiple steps in the metastatic cascade, including angiogenesis, migration, survival, and colonization at secondary organ sites. It is also required for the metastatic dissemination of cancer cells that have undergone EMT (Feng et al., 2017), and its absence inhibits tumor growth in animal models by disrupting redox homeostasis (Bobrovnikova-Marjon et al., 2010; Salaroglio et al., 2017). In this regard, Raturi et al. (2016) described that cancer cells with low levels of thioredoxin-related transmembrane protein 1 (TMX1) show increased Ca^2+^ release from the ER with a concomitant decrease in mitochondrial cation levels that results in reduction of respiration and glycolytic energy-based tumor growth (Ganapathy-Kanniappan and Geschwind, 2013). TMX1 requires thioredoxin and palmitoylation motifs to target MAM and to participate in calcium flow between ER and mitochondria contact sites (Raturi et al., 2016). Recently, the expression of FUN14 domain-containing protein 1 was positively correlated with breast cancer metastasis and the Ca^2+^/NFATC1/BMI1 axis, suggesting that inhibition of this protein could represent a therapeutic target for breast cancer (Wu et al., 2019). From the above-mentioned description, it is clear that calcium regulation is essential in carcinogenesis and tumor development. Last but not least, other MAM-tethering proteins are the mitochondrial proteins Mfn1 and Mfn2, which have been reported to be anti-proliferative and pro-apoptotic in cancer cells. Overexpression of both mitofusins in cancer cells has been demonstrated, as well as their interactions with phosphatidylinositol 3-kinase (PI3K)/Akt and P21Ras pathways, which are involved in cell proliferation, metastasis, and invasion processes (Ma et al., 2015; Li et al., 2018; Moghaddam et al., 2020).

### Lipid Exchange and Signaling in Cancer

Lipidomic remodeling, including alteration in fatty acid transport, *de novo* lipogenesis, β-oxidation, and storage as lipid droplets, is a metabolic hallmark of cancer cells (Blücher and Stadler, 2017; Enríquez-Cortina et al., 2017; Diaz-Aragon et al., 2019).

Under basal conditions, cells store fatty acids as an energy reserve to ensure their survival. They are located in bodies called *lipid droplets*, whose function is to transport lipids to all organelles (mainly to the mitochondria) (Cohen et al., 2018). Lipid transport is coupled to membrane vesicle trafficking of the secretory pathway and also supplied to different compartments through *non-vesicular traffic* (Urbani and Simoni, 1990; Lev, 2010, 2012). Lipid transfer proteins (LTPs) facilitate lipid traffic from a donor to a receptor compartment (Lev, 2010; Wong et al., 2019). Most intracellular LTPs are anchored to the MCS, whereas domains containing the lipid-binding cavity transfers the lipid cargo from one organelle to the others (Wong et al., 2017, Hanada, 2018; Wong et al., 2019). LTP dysfunction may contribute to cancer development, as it has been suggested that these proteins are involved in both migration and cell growth (Sassano et al., 2017) and that their deregulation could lead to disease progression (Peretti et al., 2020), making them an attractive target in cancer research.

LTPs acting at the ER–Golgi interface are as follows: (1) CERT, which transports the newly synthesized ceramide from the ER to the trans-Golgi network *via* the MCS (Scheffer et al., 2011; Kumagai and Hanada, 2019), (2) the four-phosphate adaptor protein 2 involved in the transport of glucosylceramide to the TGN (Mesmin et al., 2019), and (3) OSBP, which is engaged in the direct transfer of cholesterol from the ER to the TGN *via* PI4P-coupled countertransport (Mesmin et al., 2017). All LTPs share structural characteristics that allow them to bind to the Golgi and ER simultaneously, acting as a bridge. Most relevant are the N-terminal pleckstrin homology (PH) domain that is selective for PI4P in the TGN and a central motif of FFAT that binds to the VAPA and VAPB proteins located in the ER (Loewen et al., 2003; Kawano et al., 2006). In the following sections, we describe current information on the relevance of some LTPs in cancer migration and progression; in particular, we will refer to CERTs and OSBPs.

#### Ceramide Transfer Protein

CERT (encoded by *COL4A3BP*) contains a steroidogenic regulatory protein-related lipid transfer (START) terminal carboxylic domain responsible for ceramide binding and inter-membrane transfer that also shows high specificity with natural C_14__–__20_ ceramide, but not with longer acyl chains (Hanada et al., 2003; Kumagai et al., 2005).

It has been observed that stress stimuli, such as those exerted by chemotherapeutic drugs, cause an increase in ceramide levels by stimulating *de novo* ceramide synthesis, sphingomyelin hydrolysis, or both (Alphonse et al., 2004; Mesicek et al., 2010). In this sense, some tumor models are associated with impaired ceramide signaling, suggesting that this molecule plays a key role in tumor development and progression (Norris et al., 2006; Kim et al., 2008; Ruckhäberle et al., 2008). Specifically, its decrease results in resistance to cell death stimuli (Bonnaud et al., 2007), while its restoration increases sensitivity (Morales et al., 2007), which supports the central role of ceramide signaling in cell death. Swanton et al. (2007) also observed that downregulation of *COL4A3BP*, besides sensitizing cancer cells to multiple cytotoxic agents, enhances ER stress, proposing *COL4A3BP* as a possible target in chemotherapy-resistant cancers.

CERT represents the main ceramide gateway from the endoplasmic reticulum and might increase its levels in the mitochondria (Scheffer et al., 2011). In light of this, Dadsena et al. (2019) identified VDAC1 and VDAC2 channels as binding proteins to mitochondrial ceramide by using a photoactivatable ceramide probe. On the other hand, VDAC2 loss or the replacement of a membrane-facing residue of glutamate with glutamine makes human colon cancer cells resistant to ceramide-induced apoptosis (Dadsena et al., 2019). To reinforce the premise that deregulation of ceramide levels in ER is linked to mitochondrial apoptosis, Jain et al. (2017) demonstrated that mitoCERT (a ceramide transfer protein equipped with an OMM anchor) triggers mitochondrial Bax-dependent apoptosis HeLa cells.

The regulation of CERT differs between different types of cancer. For instance, CERT is overexpressed in ovarian cancer cells (Juul et al., 2010) and HER^+^ cells (Lee et al., 2012); its silencing exerts enhanced sensitivity to multi-drug treatment in several cancer cell lines (Swanton et al., 2007) and also induces changes in the levels of lysosome-associated membrane protein 2, which increases the autophagosome–lysosome flux in colorectal and breast cancer cell lines (Lee et al., 2012). On the other hand, the downward regulation of CERT improved ErbB1 mobility, ligand autophosphorylation, internalization, and chemotaxis that eventually contribute to TNBC cell progression (Heering et al., 2012). CERT decrease is also related to alterations in the activity of phospholipase D2, which facilitate the activation of HER1 (Heering et al., 2012). In this context, the activity of PLD increases in different types of cancer; for instance, it was shown that this phospholipase is dispensable for tumorigenesis and growth of breast tumors, but it is essential for lung metastasis of mammary cancer cells (Wang Z. et al., 2017).

**FIGURE 5:**
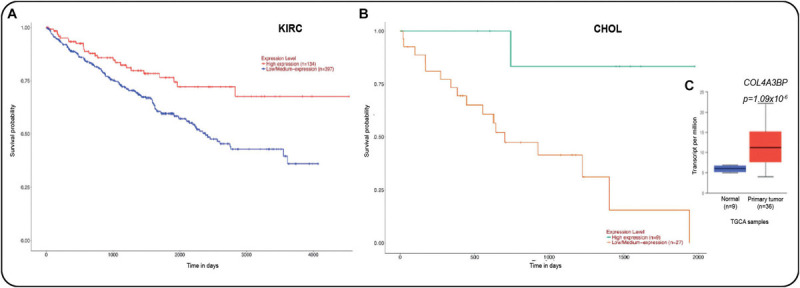
**(A)** Survival analysis for the *COL4A3BP* gene (CERT protein) in kidney renal clear carcinoma and in **(B)** in cholangiocarcinoma (CHOL). **(C)** *COL4A3BP* gene expression in CHOL samples against normal liver (*p* = 1.09 × 10^− 6^). All data were taken from the UALCAN cancer database (Chandrashekar et al., 2017).

As mentioned above, CERT1 displays a profound impact on human cancers. The survival analysis of the prediction outcome in the TCGA data set in cancer patients with high and low expression of *COL4A3BP* gene (Figure 5A) also reveals that its high expression was associated with a poor outcome in renal cancer (Figure 5A) and cholangiocarcinoma (CHOL, Figure 5B). Furthermore, cholangiocarcinoma primary tumors exhibited a higher expression of *COL4A3BP* gene as compared with normal liver (Figure 5C), according to the UALCAN database^2^ (Chandrashekar et al., 2017). The genomic findings regarding this gene in TCGA data point to it as a noteworthy target for future research, particularly in CHOL, which is the second liver primary tumor with higher mortality (Banales et al., 2020).

#### Nir

On the other hand, the Nir2 protein (which contains a PI transfer domain and transfer phosphatidic acid) (Kim et al., 2015) mediates the interactions with ER through a VAP binding site located in the Golgi. It works in coordination with CERT and OSBP in the ER–Golgi contact sites to regulate the transport of ceramide (Amarilio et al., 2005; Peretti et al., 2008). Nir2-mediated MSC allows the regulation of lipids involved in the activation of the mitogen-activated protein kinase and PI3K signaling pathways, which, in turn, play an important role in the initiation and progression of breast cancer and with EMT activation (Keinan et al., 2014; Ellis and Ma, 2019). Expression of Nir2 in MDA-MB-231 cells correlates with a poor prognosis in breast cancer. By contrast, depletion of Nir2 expression in MDA-MB-231 cells reduces metastasis in number and size (Keinan et al., 2014).

In the cell lines of colorectal adenocarcinoma SW48, the participation of Nir1 and Nir2 genes has been investigated as key regulators of cell morphogenesis. Under normal conditions, the regulation of both genes is differential, i.e., Nir1 is regulated downward, while Nir2 is regulated upward. However, these scenarios are modified with tumor suppressors such as the TGFβ-induced factor 2-linked X (TGIF2LX). In the presence/absence of TGIF2LX, Nir2 acts as a tumor suppressor and Nir1 acts as a proto-oncogene, respectively (Mobini et al., 2016). Interestingly, overexpression of TGIF2LX in C57BL/6 nude mice induced a different effect in Nir1 and Nir2, suggesting that the regulation of these genes would help suppress the progression of colorectal carcinoma (Mobini et al., 2018). Speaking of Nir1, it has been shown that its binding to chemokine ligand 18 induces the progression and metastasis of invasive ductal breast carcinoma to the lung through the LIMK/cofilin and PI3K/Akt/GSK3β/Snail signaling cascade, promoting EMT (Zhang et al., 2013). These results suggest that direct intervention on Nir1 could serve as a potential target in cancer progression, and the study of Nir2 may be useful to understand some LTP-mediated mechanisms that regulate key cellular processes and contribute to cancer metastasis.

#### Oxysterol-Binding Protein

As already mentioned, ORP proteins contain double-targeting determinants for ER and associated organelles, such as an FFAT motif that binds to VAP proteins located in the ER and PH domain that allow their interactions with different non-ER organelle membranes (Olkkonen and Li, 2013). Galmes et al. (2016) showed in HeLa cells that OPR5 and OPR8 are located in ER–mitochondria MCS, in addition to mitochondrial protein tyrosine phosphatase-interacting protein-51 that interacts with ER–PM contacts. Interestingly, the depletion of ORP5 and ORP8 leads to defects in mitochondrial morphology and respiratory function (Galmes et al., 2016).

Cholesterol has acquired great relevance thanks to its participation in carcinogenic signaling pathways (Huang et al., 2020). In this sense, it has been described that hypercholesterolemia represents a risk factor in the development of some types of cancer such as breast, prostate, liver, and colorectal cancer. At the cellular level, cholesterol is an important component of cell membranes and lipid rafts (Ding et al., 2019; Huang et al., 2020), while tumor cells use it for membrane formation during cell growth and division (Vassilev et al., 2015).

The location of cholesterol in cell membranes favors its interaction with membrane proteins. In terms of cholesterol trafficking, it has been reported that the START domain 3 (StARD3, steroidogenic acute regulatory protein-related lipid transfer domain-3) creates endosome–ER contact sites, promoting cholesterol accumulation in endosomes at the expense of the plasma membrane (Wilhelm et al., 2017). STARD3 has an N-terminal MLN64 domain (MENTAL) with four transmembrane subunits anchored to the endosome membrane, a central FFAT region, and a hydrophobic START C-terminal domain in which cholesterol binds (Voilquin et al., 2019). This process is favored by the interaction with ER-anchored VAP protein, generating a highly efficient cholesterol transport (Wilhelm et al., 2017). Overexpression of StARD3 was identified in at least 14 of 93 invasive breast carcinomas, which also expressed high levels of HER2 mRNA, and may increase oncogenic signaling through membrane-associated kinases such as proto-oncogene tyrosine-protein kinase Src (Vassilev et al., 2015). In this regard, Zhang et al. (2011) evaluated the activation of Src in primary breast tumor samples of patients treated with trastuzumab (an anti-HER2 monoclonal antibody indicated for the treatment of tumors that overexpress HER2) by immunohistochemical staining and demonstrated a strong correlation between patients expressing high levels of Src activity and low survival as well as high survival in those patients with low Src activity (Zhang et al., 2011), suggesting that STARD3 may contribute to the aggressive behavior of patients’ resistance to trastuzumab.

It has also been described that STARD3, when overexpressed, generates rigid endosome and a static ER–endosome contact site, preventing the late maturation of endosome to lysosome and consequently blocking lysosomal degradation of cell surface receptors (including HER2 and other growth factors). In turn, the receptors are recycled back to the plasma membrane, favoring the spread of cell signals of uncontrolled cell growth. Thus, STARD3 increases the progression of HER2-positive cancer (Peretti et al., 2020). Qiu et al. (2014) reported a strong expression of STARD3 in tubular adenocarcinoma cells, positively associated with a high mitochondria number. In this sense, alterations in mitochondrial cholesterol trafficking have been associated with inhibition of cell death by inhibiting the release of cytochrome c and Smac/Diablo, facilitating the survival of tumor cells (Smith and Land, 2012). Interestingly, even in non-cancerous cells, cholesterol accumulation in hepatocytes is strongly associated to apoptosis resistance and mitochondrial dynamic changes (Domínguez-Pérez et al., 2019). Mitochondrial cholesterol is also associated with resistance to chemotherapy. Montero et al. (2008) found that StART silencing in H35 and HepG2 cells significantly decreases cholesterol levels in the mitochondria as a result of cholesterol delivery reduction from extramitochondrial sources into the mitochondria. Additionally, over-accumulation of cholesterol in tumor tissues is strongly associated with low serum cholesterol levels in patients diagnosed with cancer (Strasak et al., 2009; Benn et al., 2011; Smith and Land, 2012). Finally, it has been reported that mitochondrial cholesterol overload is directly associated with aggressive liver cancer phenotype with poor prognosis (Enríquez-Cortina et al., 2017). In brief, LTPs impact cancer progression and metastasis; however, more studies are needed to understand the specific function of LTPs.

In the next section, we tackle the effect of cancer chemotherapy and its relationship with MCS, with greater emphasis on ER–mitochondria contact sites and calcium management, which are the most reported players in cancer progression and metastasis.

## MCS as Possible Therapeutic Targets in Cancer

Although pioneering studies by Howatson and Ham (1955) reported a limited number of contacts between the ER and the mitochondria in rat liver cancer compared to normal tissue, it was the first evidence of the relationship between organelles and their importance in tumorigenesis. As it is known, organelles sense stress in the cellular microenvironment and modify their structure and function according to cellular demand for survival; however, deterioration of the signaling cascades might lead to oncogene activation. In this regard, oncoproteins located in the MAM, such as AKT (Betz et al., 2013), PERK (Fels and Koumenis, 2006; Bu and Diehl, 2016), Grp75 (Wadhwa et al., 2006), and VDAC (Pernemalm et al., 2013; Shoshan-Barmatz et al., 2015) as well as tumor suppressors such as p53 (Giorgi et al., 2015), the phosphatase tensin homolog (Bononi et al., 2013), and the promyelocytic leukemia (Giorgi et al., 2010; Missiroli et al., 2016), interact with Ca^2+^ handling proteins, modulating their activity and promoting tumorigenesis (Morciano et al., 2018; Xia et al., 2019).

Anti-cancer drugs have multiple modes of action; in this regard, much attention has been paid to calcium signaling, as its deregulation in MAMs has been identified as a hallmark of cancer cells. Ca^2+^ ions shift cancer metabolism toward glycolytic metabolism and increase their resistance to cell death (Bittremieux et al., 2016). These also support mesenchymal transformation, migration, invasion, and metastasis (Ren et al., 2017). However, changes in Ca^2+^ signaling depend on the type of cancer cell and the chemotherapeutic treatment used. For instance, *cis*-platin (used for solid tumors) in rat dorsal root ganglion neurons blocks VDAC at high concentrations (Tomaszewski and Büsselberg, 2007), while in prostate cancer it promotes calcium uptake through other channels (Chen et al., 2014) and in lung carcinoma it favors its liberation *via* SOCE (Gualdani et al., 2019). Therefore, management of Ca^2+^ signaling is a major complication in chemotherapeutic treatments (Varghese et al., 2019).

There is a growing number of reports on Ca^2+^ signaling alterations in MCS research, but the information in this area still remains scarce. The following sections present current evidence on the role of conventional chemotherapeutic strategies and their relevance in MCS as a study target in cancer treatment.

### Chemotherapeutic Compounds and Their Role in MCS

Some compounds used in chemotherapy have been shown to indirectly modulate interactions between the ER and the mitochondria. However, there are no conclusive studies that demonstrate their direct participation. It has been suggested that these drugs could act on some of the MCS’s interaction proteins. For example, the expression of Mfn2 (which controls the stability of the ER–mitochondria interaction and the transfer of Ca^2+^ and lipids) decreases in neonatal cardiomyocytes exposed to doxorubicin (Dox) (Tang et al., 2017). Other reports indicate that p53, located in the ER, MAM, and cytosol under basal conditions, is accumulated in the ER–mitochondria interface in response to Dox in *p53^+/+^* HTC-116 cells, modulating Ca^2+^ homeostasis. In this sense, p53 interacts with SERCA, promoting the accumulation of calcium in the ER and its subsequent transfer to the mitochondria, triggering apoptosis (Giorgi et al., 2015). Betz et al. (2013) showed that mTORC2 is detected in the contacts of the mitochondria with the ER where it phosphorylates and activates the Akt after growth factor stimulation. In turn, mTORC2–Akt signaling regulates MAM integrity, Ca^2+^ flux in the ER–mitochondria contacts, and energy metabolism. Nevertheless, some of the side effects of Dox are related to Ca^2+^. Aziz et al. (2019) reported that calcium derived from the ER increases significantly as compared to cytosolic calcium via Src kinase activation in rat ovarian follicles (Aziz et al., 2019).

*cis*-Platin can also affect MCS homeostasis. Studies in the A549 cell lines resistant to *cis*-platin show elevated concentrations of free intracellular Ca^2+^ (Liang and Huang, 2000). Other studies showed that the IP_3_R gene is deregulated in the drug-resistant cell, explaining in part the bladder cancer cells’ resistance to apoptosis (Tsunoda et al., 2005). Xu et al. (2015) also revealed that *cis*-platinum causes the pro-apoptotic release of Ca^2+^ from the ER in the cytosol and the mitochondria, leading to cationic overload in both compartments and ultimately contributing to ER- and mitochondria-mediated apoptosis in *cis*-platin-sensitive SKOV3 cells (Xu et al., 2015). The same group demonstrated that ABT737 (a pharmacological Bcl-2 inhibitor that interacts with IP_3_R and suppresses Ca^2+^ signaling) (Akl et al., 2014) increases *cis*-platinum cytotoxicity in chemically resistant SKOV3 cells. Interestingly, ABT737 increased the free calcium levels both in the cytosol and the mitochondria, accentuating apoptosis mediated by the ER–mitochondria contacts (Xie Q. et al., 2016). On the other hand, BIRD2 inhibits Bcl-2/IP_3_R interaction by attenuating Bcl-2 control after Ca^2+^ elevation and calcium-mediated apoptosis in different types of cancer *in vitro* and *in vivo* (Distelhorst, 2018). Bittremieux et al. (2019) recently showed that BIRD2 changes calcium signals from pro-survival to pro-death in the cancer cell, that is, IP_3_R-mediated calcium release from the ER causes a marked increase in intracellular Ca^2+^, leading to calcium mitochondrial overload and apoptosis triggering (Bittremieux et al., 2019).

In addition, chemotherapeutic compounds are related to some of the cellular processes that take place at the MCS interface. For instance, in pancreatic cancer cells, *cis*-platin stimulates ER stress and interacts with bortezomib (a potent and selective proteome inhibitor for the treatment of solid neoplasias) by increasing ER dilatation, [Ca^2+^]_i_ levels, and cell death. It is important to note that combination therapy (bortezomib plus *cis*-platin) induces JNK activation and apoptosis in these cells, resulting in a reduction in tumor burden and suggesting that this combination increases the anti-cancer activity of *cis*-platin (Nawrocki et al., 2005). On the other hand, oxaliplatin (a platinum-based drug) acts as a mediator of Ca^2+^ signaling and plays a role in peripheral neuropathy. It has been described that prolonged exposure to the drug induces changes in ER loading and IP_3_R-mediated Ca^2+^ signaling in SH-SY5Y neuroblastoma cells (Schulze et al., 2011).

Most of the works cited here refer to different cancer cell lines, where the protection mechanism of pharmacological compounds is associated with calcium management. However, the relevance of these molecules at the interface of the ER–mitochondria contact sites, as well as in other interactions in cancer, is still scarce; we believe that it is paramount to study not only the management of Ca^2+^ but also other processes carried out in the MCS that could be modulated by some of the molecules already described.

## Conclusion and Perspectives

The identification of new targets that provide better prognosis in cancer is necessary due to the increase in the number of new cases in the world. In particular, the increased interest in the study of contact sites in different physiopathological conditions suggests that they could be placed as novel targets in cancer studies. However, even though several working groups are actively studying the role of MCS in cancer (as well as in other pathological conditions), there are missing links that must be incorporated to understand their function in an integrated way.

**FIGURE 6:**
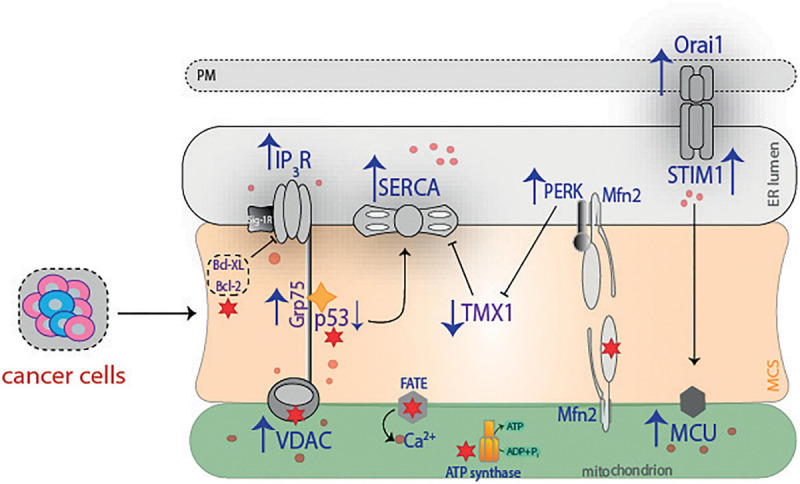
Summary of proteins with relevance in cancer. The progression and metastasis of cancer cells benefit is associated with an overexpression of different proteins. To mention a few examples, IP_3_ overexpression favors cancer migration, with a concomitant increase in Ca^2+^ concentrations, while VDAC and SERCA increase is related with poor prognosis. Grp75 interacts with p53 and promotes cancer cell survival, whereas Orai1 and STIM1 boost Ca^2+^ and allow cancer cell migration. On the other hand, PERK increases invasion and metastasis by limiting redox homeostasis through TMX1, which is decreased in cancer. Ultimately, red stars illustrate the effect that different therapies exert on cancer. PM, plasma membrane; STIM1, stromal interaction molecule 1; IP_3_R, inositol 1,4,5-triphosphate receptor; SERCA, sarco(endo)plasmic reticulum Ca^2+^-ATPase; PERK, protein kinase RNA-like ER kinase; Mfn2, mitofusin 2; Bcl-XL, B-cell lymphoma—extra large; Bcl-2, B cell lymphoma-2; Grp75, glucose-related protein 75; p53, tumor suppressor protein; TMX1, thioredoxin-related transmembrane protein 1; VDAC, voltage-dependent anion channel; ATP, adenosine triphosphate; ADP, adenosine diphosphate; MCU, mitochondrial calcium uniporter; MCS, membrane contact sites.

The nutraceutical compounds’ effect on contact sites is also an interesting issue to be explored. The relevance of these molecules lies in their ability to regulate some of the proteins that conform with the MCS machinery, such as Mfn2, IP_3_R, VDAC1, but they could also function as adjuvant therapy to improve the beneficial effects of conventional therapy (Figure 6). It is clear that a considerable number of proteins that regulate membrane contacts have been “discovered” in recent years, but it has to be determined which of them might have the potential to reduce cancer progression. Overall, one of the most attractive and immediate perspectives in the field of “contactology” (Csordás et al., 2018) would be to elucidate and/or describe what other types of organelle interactions occur in cancer as well as to detail how they would favor and/or inhibit cancer progression. Undoubtedly, it remains a real challenge to limit the progression of cancer, but great advances in this area have been made. We believe that understanding the MCS mode of action could improve the management of several diseases.

Finally, we believe that more studies of MCS might position them as novel relevant markers for diagnostic and prognostic purposes in cancer.

## Author Contributions

AG-H, MA-C, CZ, LEG-Q, and AS-P have made a substantial, direct, and intellectual contribution to the work. AS-N and LEG-Q have made the bioinformatic analysis used in this manuscript. AS-P authorized and reviewed the drafts of the document. All authors approved its publication.

## Conflict of Interest

The authors declare that the research was conducted in the absence of any commercial or financial relationships that could be construed as a potential conflict of interest.

## Footnotes

1 https://www.cancer.gov/

2 http://ualcan.path.uab.edu
